# Lipid-Free Antigen B Subunits from *Echinococcus granulosus*: Oligomerization, Ligand Binding, and Membrane Interaction Properties

**DOI:** 10.1371/journal.pntd.0003552

**Published:** 2015-03-13

**Authors:** Valeria Silva-Álvarez, Gisela R. Franchini, Jorge L. Pórfido, Malcolm W. Kennedy, Ana M. Ferreira, Betina Córsico

**Affiliations:** 1 Instituto de Investigaciones Bioquímicas de La Plata (INIBIOLP) (UNLP-CONICET), Facultad de Ciencias Médicas, Universidad Nacional de La Plata (UNLP), La Plata, Argentina; 2 Consejo Nacional de Investigaciones Científicas y Técnicas (CONICET), Ciudad Autónoma de Buenos Aires, Argentina; 3 Institute of Molecular, Cell and Systems Biology, College of Medical, Veterinary and Life Sciences, University of Glasgow, Glasgow, Scotland, United Kingdom; 4 Cátedra de Inmunología, Facultad de Ciencias/Facultad de Química, Universidad de la República (UdelaR), Montevideo, Uruguay; University of Würzburg, GERMANY

## Abstract

**Background:**

The hydatid disease parasite *Echinococcus granulosus* has a restricted lipid metabolism, and needs to harvest essential lipids from the host. Antigen B (EgAgB), an abundant lipoprotein of the larval stage (hydatid cyst), is thought to be important in lipid storage and transport. It contains a wide variety of lipid classes, from highly hydrophobic compounds to phospholipids. Its protein component belongs to the cestode-specific Hydrophobic Ligand Binding Protein family, which includes five 8-kDa isoforms encoded by a multigene family (*EgAgB1-EgAgB5*). How lipid and protein components are assembled into EgAgB particles remains unknown. EgAgB apolipoproteins self-associate into large oligomers, but the functional contribution of lipids to oligomerization is uncertain. Furthermore, binding of fatty acids to some EgAgB subunits has been reported, but their ability to bind other lipids and transfer them to acceptor membranes has not been studied.

**Methodology/Principal Findings:**

Lipid-free EgAgB subunits obtained by reverse-phase HPLC were used to analyse their oligomerization, ligand binding and membrane interaction properties. Size exclusion chromatography and cross-linking experiments showed that EgAgB8/2 and EgAgB8/3 can self-associate, suggesting that lipids are not required for oligomerization. Furthermore, using fluorescent probes, both subunits were found to bind fatty acids, but not cholesterol analogues. Analysis of fatty acid transfer to phospholipid vesicles demonstrated that EgAgB8/2 and EgAgB8/3 are potentially capable of transferring fatty acids to membranes, and that the efficiency of transfer is dependent on the surface charge of the vesicles.

**Conclusions/Significance:**

We show that EgAgB apolipoproteins can oligomerize in the absence of lipids, and can bind and transfer fatty acids to phospholipid membranes. Since imported fatty acids are essential for *Echinococcus granulosus*, these findings provide a mechanism whereby EgAgB could engage in lipid acquisition and/or transport between parasite tissues. These results may therefore indicate vulnerabilities open to targeting by new types of drugs for hydatidosis therapy.

## Author Summary

*Echinococcus granulosus* is a causative agent of hydatidosis, a parasitic disease that affects humans and livestock with significant economic and public health impact worldwide. Antigen B (EgAgB), an abundant product of *E*. *granulosus* larvae, is a lipoprotein that carries a wide variety of lipids, including fatty acids and cholesterol. As *E*. *granulosus* is unable to synthesize these lipids, EgAgB likely plays an important role in parasite metabolism, participating in both the acquisition of host lipids and their distribution between parasite tissues. The protein component of EgAgB consists of 8 kDa subunits encoded by separate genes. However, the biochemical properties of EgAgB subunits, particularly their ability to bind and transfer lipids, are poorly known. Herein, using *in vitro* assays, we found that EgAgB subunits were capable of oligomerizing in the absence of lipids and to bind fatty acids, but not cholesterol. Moreover, EgAgB subunits showed the ability to transfer fatty acids to artificial phospholipid membranes. These results indicate new points of attack at which the parasite might be vulnerable to drugs.

## Introduction

Cystic echinococcosis (CE), one of two major types of hydatid disease, is a worldwide zoonosis caused by the larval stage (metacestode) of *Echinococcus granulosus* sensu lato (*E*. *granulosus* s.l.), which includes a series of species traditionally considered to comprise different strains or genotypes of *E*. *granulosus* [1,2]. The larva forms unilocular bladder-like cysts (referred to as hydatid cysts) that establish and gradually grow within the viscera (mainly liver and lungs) of a wide range of mammalian species (mainly domestic ungulates) as well as humans [1]. CE is considered a chronic, complex and neglected disease, which is re-emerging as an important public health problem [3–6]. For many years surgery was considered the only effective therapy, although it is not recommended for patients with cysts disseminated into different organs, and had a relatively high morbidity, relapse and mortality rates [7,8]. Currently, the advent of antihelminthic drugs (benzimidazole carbamates, mainly mebendazole and albendazol) has led to an alternative therapy which comprises pre- and post-operative chemotherapy, combined with percutaneous drainage of hydatid cysts (a procedure known as PAIR for puncture, aspiration, injection, reaspiration) [7,8]. In comparison with surgery this approach showed greater clinical and anti-parasitic efficacy (lower rates of morbidity, mortality, and disease recurrence and shorter hospital stays [9]). In addition, antihelminthic drugs are chosen for the treatment of uncomplicated cysts, as well as for long-term post-surgical treatment [8]. Benzimidazoles bind to β-tubulin and interfere with microtubule formation, thus affecting motility, cell division, secretion processes, as well as perturbing the uptake of glucose by helminths [10,11]. Nevertheless, benzimidazole treatment has shown limited efficacy against large cysts and the occurrence of side-effects has also been reported [12]. Therefore, the development of novel drugs against *E*. *granulosus* s.l. therapy is required. Advancing knowledge of parasite biology would facilitate the identification of new drug targets for a more specific CE therapy.

Antigen B (EgAgB) is an abundant lipoprotein of hydatid cyst fluid that has been postulated as a carrier of essential lipids for *E*. *granulosus* s.l. [13–16]. This is based on the fact that cestodes have lost both degradative and biosynthetic pathways for common fatty acids and sterols [17,18], and EgAgB contains a heterogeneous mixture of lipids including free and esterified fatty acids and sterols [15]. Thus, EgAgB may be of foremost importance for parasite lipid metabolism, representing an interesting target for chemotherapy.

**Fig 1:**
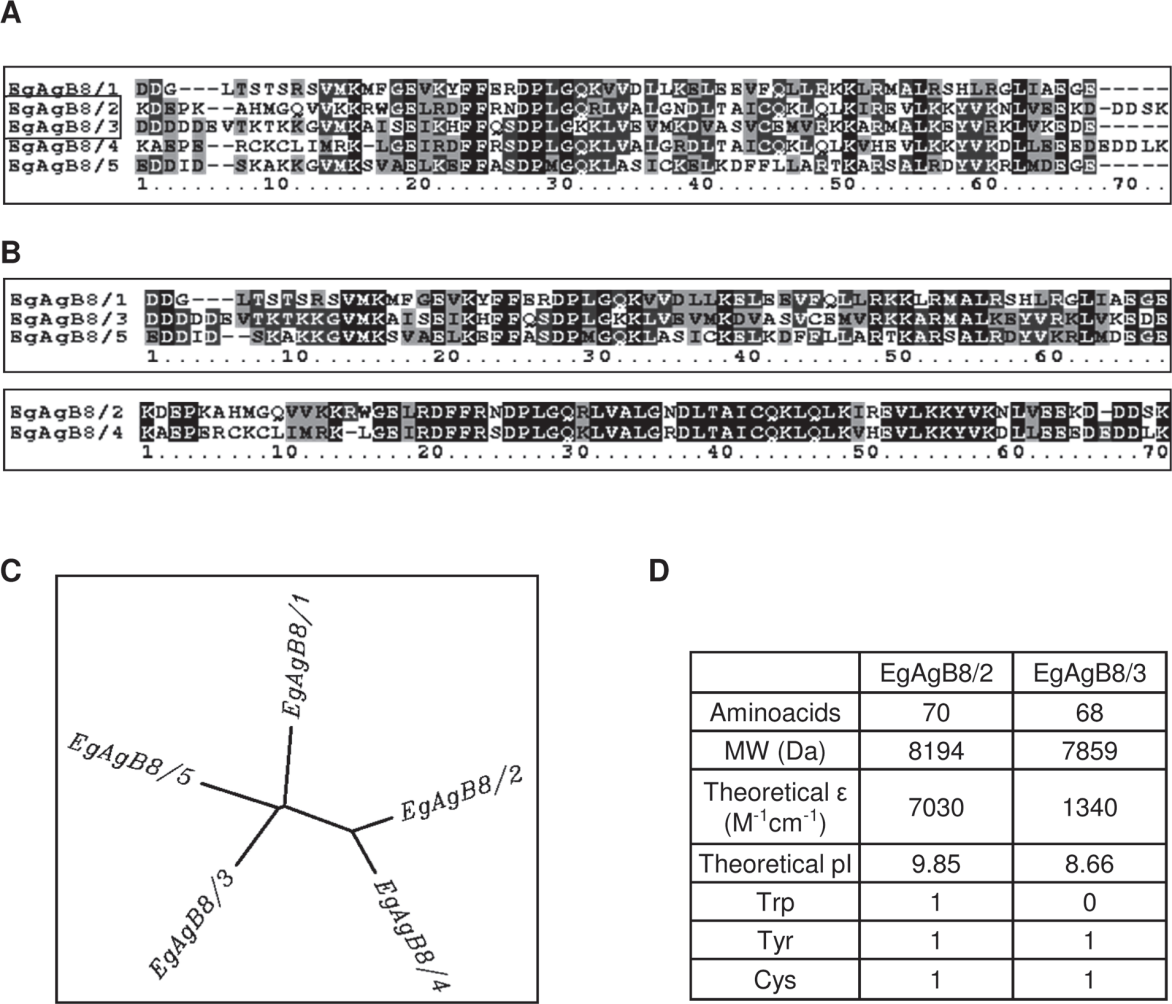
Amino acid sequence analysis of EgAgB8 subunits. Analysis of EgAgB8 subunits were undertaken using Biology Workbench 3.2 (University of Illinois, National Center for Supercomputing Applications, USA). **(A)** Alignment of amino acid sequences of the mature peptides of EgAgB8/1, EgAgB8/2, EgAgB8/3, EgAgB8/4 and EgAgB8/5, using *ClustalW* from Biology Workbench 3.2. Absolutely conserved residues, partially residues and similar residues are shown in black, dark grey and light grey, respectively. Comparison between EgAgB8 mature peptides shows that EgAgB8/1, EgAgB8/3 and EgAgB8/5 are more closely related to each other than to EgAgB8/2 and EgAgB8/4, as shown in the alignment **(B)** and in the tree **(C)** obtained employing *DrawTree* from Biology Workbench 3.2. **(D)** Biochemical data for EgAgB8/2 and EgAgB8/3 (from sequences squared in A). The theoretical isoelectric point (pI) and the theoretical extinction coefficient were also estimated using Biology Workbench 3.2 software. Accession numbers: EgAgB8/1: AAD38373, EgAgB8/2: AAC47169, EgAgB8/3: AAK64236, EgAgB8/4: AAQ74958 and EgAgB8/5: BAE94835.

EgAgB is an alpha helix-rich 230 kDa lipoprotein [15], which has been considered to be the most specific *Echinococcus*-genus antigen for human serodiagnosis of hydatid disease [19–21] as well as being an immunomodulatory parasite component [22,23]. It belongs to a cestode-specific family of proteins that bind hydrophobic ligands, referred to as hydrophobic ligand binding proteins (HLBPs). The ligands present in the EgAgB complex account for approximately 40–50% of the total mass of the native antigen and consist of a variety of neutral (mainly triacylglycerides, sterols and sterol esters) and polar (mainly phosphatidylcholine) lipids [15]. At the protein level, native EgAgB contains around a dozen apolipoproteins or subunits [15], which are encoded by a polymorphic multigene family comprising five clades named *EgAgB*1 to *EgAgB5* [24–29]. Interestingly, EgAgB isoforms are expressed differentially during the life-cycle stages of the parasite, as well as within distinct tissues of a given developmental stage; *EgAgB1* to *EgAgB4* are expressed in the metacestode stage whereas *EgAgB5* seems to be expressed in the adult stage. Furthermore, in the metacestode, *EgAgB1* to *EgAgB4* are expressed in the germinal layer, but *EgAgB3* seems to be the most abundantly expressed in protoscoleces [29]. Similar evidence of differential expression of antigen B subunits has also been obtained for the closely related species *E*. *multilocularis* [30]. The proteins encoded by EgAgB genes are each approximately 8 kDa in mass, and the different isoforms designated EgAgB8/1 to EgAgB8/5. Comparison of their amino acid sequences shows that EgAgB8/1, EgAgB8/3 and EgAgB8/5 are more similar to each other than to EgAgB8/2 and EgAgB8/4 (Fig. 1).

One of the features of EgAgB subunits is their ability to self-associate into large complexes. Analysis of native EgAgB from hydatid cyst fluid showed oligomers of 16, 24 and 32 kDa that are built from the 8 kDa subunits [31], as found in SDS-PAGE analysis under reducing conditions [32]. Recombinant subunits of EgAgB8/1, EgAgB8/2 and EgAgB8/3 are also capable of self-associating into oligomers of 16 and 24 kDa, and also into high-order oligomers of more than 100 kDa, estimated by size exclusion chromatography [33] or native polyacrylamide gel electrophoresis [34]. These results indicate that recombinant EgAgB subunits share structural features with native EgAgB, which agrees with the fact that EgAgB subunits present an electrostatic profile compatible with molecular aggregation [16,33] and suggest that lipids may not be indispensable for oligomerization. Nevertheless, the contribution of the lipid moiety to the oligomerization process has not yet been formally considered.

As already mentioned, EgAgB particles contain a mixture of lipid classes ranging from highly hydrophobic lipids to a variety of phospholipids [15]. The lipid binding properties of different members of EgAgB family have been partially characterised and constitute a piece of information that may contribute to elucidate EgAgB functions. EgAgB8/1 and EgAgB8/2 ligand binding properties were analysed by Chemale and collaborators, who used recombinant subunits (delipidated using the hydrophobic resin Lipidex 1000) and fluorescent probes to examine their interaction with fatty acids [35]. They observed that both subunits were capable of binding the fatty acid analogue 16-AP (16-(9-anthroyloxy) palmitate), but not the fluorescent probes DAUDA (11-(dansylamino) undecanoic acid), ANS (1-anilinonaphthalene-8-sulfonic acid) or DACA (dansyl-DL-alpha-aminocaprylic acid). In these studies, EgAgB8/1 and EgAgB8/2 were found to bind fatty acids with similar affinity. However, differences in the ability to bind lipids among other EgAgB members could not be ruled out. In fact, the 150 kDa HLBP entity of *Taenia solium* (TsM150) contains two classes of HLBP subunits (7 and 10 kDa members) which are capable of binding different lipid probes; members of the 7kDa-TsM150 subfamily bind DAUDA, ANS and DACA, but not 16-AP, whereas members of the 10kDa-TsM150 subfamily only bind 16-AP [36]. Furthermore, analysis of the ability of EgAgB subunits to bind lipid classes different from fatty acids has not been analysed, which, considering the biochemical composition of native EgAgB-associated lipids [15], is a potentially serious gap in our knowledge.

As previously stated, the fact that EgAgB carries lipids that are essential to *E*. *granulosus* s. l. suggests a role for EgAgB in the uptake and delivery of these lipids [37]. If so, then EgAgB would be expected to transfer ligands between donor and acceptor membranes and/or membrane-embedded receptor proteins. The ability of EgAgB to transfer ligands or to interact with membranes has not yet been examined in detail.

In the present work, we report a novel method of preparing lipid-free EgAgB8 subunits, which allowed the proper analysis of their oligomerization capacity and lipid binding properties. For this purpose, we purified the recombinant subunits EgAgB8/2 and EgAgB8/3 as representatives of the two distinct types of subfamilies within the multigenic EgAgB family (Fig. 1), and then removed their co-purifying ligands (mainly phospholipids) by reverse-phase high performance liquid chromatography (RP-HPLC). We found that lipid-free EgAgB8/2 and EgAgB8/3 subunits were able to self-associate into larger oligomers, suggesting that lipids are not indispensable for oligomerization. Regarding their lipid binding properties, both subunits bound a stearic acid anthroyloxy-derivative with similar affinity, but not a fluorescent cholesterol analogue. Furthermore, using small unilamellar vesicles we found that EgAgB8/2 and EgAgB8/3 subunits are potentially capable of transferring fatty acids analogues to phospholipid membranes employing different mechanisms, in which electrostatic interactions might play an important role. Overall, these results indicate previously unsuspected features of EgAgB particle assembly and suggest interaction with cell membranes (possibly of both parasite and host) that present potentially new classes of therapeutic target.

## Materials and Methods

### Materials

Inorganic salts were acquired from Sigma Chemicals (USA), Merck (Germany) or Carlo Erba (France). Organic solvents were purchased from JT Baker (USA), Merck (Germany) or Carlo Erba (France). For purification and delipidation of recombinant EgAgB8 subunits Gluthathione Sepharose 4B resin was obtained from GE Healthcare Life Sciences (Sweden), reduced glutathione and thrombin from human plasma were purchased from Sigma Chemicals (USA) and C8-bonded silica column was purchased from Vydac (USA). For lipid analysis silica TLC plates (20 x 20 cm) were obtained from Merck (Germany). For size exclusion chromatography (SEC) experiments, Superdex 200 HR 10/30 column was from GE Healthcare Life Sciences (Sweden), bovine serum albumin (BSA), carbonic anhydrase and cytochrome c were acquired from Sigma Chemicals (USA). For crosslinking experiments, N-ethyl-3-(3-dimethylaminopropyl)-carbodiimide (EDC) was purchased from Sigma Chemicals (USA). For ligand binding assays, fatty acid analogues 12-(9-anthroyloxy) stearic acid (12-AS) and DAUDA were obtained from Molecular Probes (USA), whereas cholesterol analogue dehydroergosterol (DHE) was obtained from Sigma Chemicals (USA). For ligand transfer assays, egg phosphatidylcholine (EPC), brain phosphatidylserine (PS), heart cardiolipin (CL) and N-(7-nitro-2,1,3-benzoxadiazol-4-yl)-phosphatidylcholine (NBD-PC),) were purchased from Avanti Polar Lipids (USA).

### Purification and delipidation of recombinant subunits of EgAgB8

Genes encoding EgAgB8/2 and EgAgB8/3 subunits subcloned into pGEX plasmids were generously donated by Dr. Arnaldo Zaha (Federal University of Rio Grande do Sul, Brazil). EgAgB8/2 and EgAgB8/3 gene sequences were confirmed employing Macrogen sequencing facility (Macrogen Inc., Korea). EgAgB recombinant subunits EgAgB8/2 and EgAgB8/3 were expressed in *Escherichia coli BL21 Codon Plus pRIL*, as glutathione S-transferase (GST) fusion proteins, purified by affinity chromatography on immobilized glutathione and recovered by thrombin cleavage as previously described [38]. The removal of hydrophobic ligands derived from the bacterial expression system was achieved by RP-HPLC in a HPLC System (Merck-Hitachi, Japan) with a C8-bonded silica as stationary phase and water/acetonitrile/trifluoroacetic acid mobile phase, based on a procedure described by Meenan and collaborators to delipidate other lipid binding proteins (LBPs) [39]. After elution and freeze drying, proteins were refolded in a large volume of phosphate buffered saline, pH 7.4 (PBS) and then concentrated using centrifugal filter units (Millipore EMD). Proper delipidation of recombinant subunits was controlled by analysing the lipid content of protein fractions subjected and not-subjected to RP-HPLC method. Lipids of pre and post-HPLC fractions were extracted using the Folch method [40], analysed by thin layer chromatography (TLC) and compared with standards, as previously described for the analysis of the lipid moiety of native EgAgB [15]. Sodium dodecyl sulfate-polyacrylamide gel electrophoresis (SDS-PAGE, 15%) followed by Coomassie blue staining was used to assess subunits purity [41]. Protein concentration was estimated by measuring the absorbance at 280 nm, employing molar extinction coefficients of 7030 and 1340 M^−1^cm^−1^ for EgAgB8/2 and EgAgB8/3, respectively (calculated from their amino acid sequences employing Biology Workbench 3.2 free software [Computational Biology Group, Department of Bioengineering, University of California, San Diego]).

### Circular dichroism spectroscopy

Lipid-free EgAgB8 subunits were analysed by circular dichroism (CD) spectroscopy to examine their structure. CD spectra of EgAgB8/2 and EgAgB8/3 (30 μM) at 25°C in PBS were recorded on a Jasco J-810 spectropolarimeter. Data in the Far-UV (195–250 nm) region were collected in 1-mm path cuvettes using a scan speed of 20nm/min with a time constant of 1 second. Molar ellipticity [θ] (deg cm^2^ dmol^−1^) was calculated as described elsewhere [42]. Secondary structure calculations of EgAgB subunits were undertaken employing *k2d* program (http://kal-el.ugr.es/k2d/k2d.html) [43,44].

### Size exclusion chromatography

SEC experiments were carried out in an Âkta FPLC System (GE Healthcare Life Sciences, Sweden). Briefly, 100 μL of lipid-free EgAgB8/2 (324 μM) or EgAgB8/3 (320 μM) were loaded separately on a Superdex 200 HR 10/30 column equilibrated in PBS. A flow rate of 0.5 mL/min was used, and elution profiles were recorded following the UV absorption at 215 and 280 nm. The column was calibrated using bovine serum albumin (66 kDa), carbonic anhydrase (29 kDa) and cytochrome c (12 kDa) as protein standards under the same chromatography conditions. Samples, as well as standard proteins, were analysed twice under the same conditions. Molecular weight estimation of the proteins was undertaken as described previously [45]. In order to investigate the stability of the oligomeric EgAgB complexes, serial dilutions of the proteins were also loaded under the same conditions.

### Cross-linking experiments

Cross-linking with EDC was carried out by adding a fresh solution of EDC to 30 μM lipid-free EgAgB8 subunits in PBS up to a final concentration of 5, 10 and 20 mM EDC. Controls without adding EDC were carried out under the same conditions at the same time. The mixture was incubated for 30 minutes at 25°C under continuous stirring. The reaction was stopped by adding SDS-PAGE sample buffer, the reaction products were analysed on a 15% SDS-PAGE followed by silver staining. In parallel, non-delipidated recombinant EgAgB8 subunits, and native EgAgB (purified from hydatid cysts as previously described [15]) were used for comparison. In addition, cross-linking experiments were performed using a non-related lipid binding protein (ABA-1-A1 from *Ascaris suum*) as a control.

### Ligand-binding assays

Fluorescence measurements were performed at 25°C in a Fluorolog-3 Spectrofluorometer (Horiba-Jobin Yvon) using 2 mL samples in a quartz cuvette. Fluorescent probes DHE, DAUDA and 12-AS were used to determine lipid binding properties of EgAgB8/2 and EgAgB8/3. Briefly, 0.5 μM of these probes were incubated at 25°C for 3 min in buffer 40 mM Tris, 100 mM NaCl, pH 7.4 (TBS) with increasing concentrations of EgAgB8 subunits. Emission spectra were recorded at 350–515, 365–665 or 400–500 nm, employing an excitation wavelength of 325, 345 and 383 nm for DHE, DAUDA and 12-AS, respectively. For 12-AS titration curves, fluorescence data were fitted using SigmaPlot software and different fittings were tested. The “one site saturation ligand binding” model showed the best *r* values (*r ∼ 0*.*99*), and was thus selected to estimate the binding constants (K_d_). Average values for three independent experiments are reported. Changes in the intrinsic tryptophan (Trp-derived) fluorescence of EgAgB8/2 (5 μM or 2 μM) were monitored upon the addition of oleic acid (0.5 to 6.5 μM) or cholesterol (0.7 to 19.4 μM). The emission spectra were recorded at 310–400 nm, employing an excitation wavelength of 295 nm.

### Vesicle preparation

Small unilamellar vesicles (SUV) were prepared by sonication and ultracentrifugation as described previously [46]. The standard vesicles were prepared to contain 90 mol % of EPC and 10 mol % of NBD-PC, which served as the fluorescent quencher of the anthroyloxy-derivative. To increase the negative charge density of the acceptor vesicles, either 25 mol % of PS or CL was incorporated into the SUVs replacing an equimolar amount of EPC. Vesicles were prepared in TBS except for SUV containing CL which were prepared in TBS with 1 mM EDTA.

### Partition coefficient

The relative partition coefficient (K_p_) of 12-AS between EgAgB and NBD-containing SUVs was determined employing a method described by Massey and collaborators [47]. The K_p_ was defined as:

$${\text{K}}_{\text{p}}=\frac{\left[{12\text{AS}}_{\text{EgAgB}}\right]}{\left[\text{EgAgB}\right]}\times \frac{\left[\text{SUV}\right]}{\left[{12\text{AS}}_{\text{SUV}}\right]}$$ (1)

where [12AS_EgAgB_] and [12AS_suv_] are the concentrations of 12-AS bound to EgAgB and vesicles, respectively, and [EgAgB] and [SUV] are the concentrations of protein and vesicles. The K_p_ for 12-AS partitioning was determined by measuring 12-AS fluorescence (440 nm) at different molar ratios of SUV:EgAgB after addition of SUVs into a solution containing 7.5 μM EgAgB8/2 or EgAgB8/3 and 0.5 μM 12-AS in buffer TBS at 25°C. The K_p_ was calculated by fitting the equation described by De Gerónimo and collaborators [48] to our data, as follows:

$${\text{F}}_{\text{rel}}=\frac{\text{a}\times {\text{K}}_{\text{p}}}{{\text{K}}_{\text{p}}+\frac{\left[\text{SUV}\right]}{\left[\text{EgAgB}\right]}}-\text{b}\times \frac{\left[\text{SUV}\right]}{\left[\text{EgAgB}\right]}$$ (2)

where Frel is the difference between the fluorescence of 12-AS at a given SUV:EgAgB ratio and the fluorescence of 12-AS with an excess of vesicles, relative to the maximum difference in 12-AS fluorescence; [SUV] and [EgAgB] are the molar concentrations of SUV and EgAgB, respectively; and “a” and “b” are the fitting parameters. The partition coefficient was used to establish the conditions, for the 12-AS transfer assay, that ensure essentially unidirectional transfer, as detailed below.

### Ligand transfer assays

A Förster Resonance Energy Transfer (FRET) assay was used to monitor the transfer of 12-AS from EgAgB to acceptor model membranes as described previously for other LBPs [49–56]. All the transfer experiments were conducted employing a Stopped-Flow RX2000 module (Applied Photophysics) attached to the spectrofluorometer. Transfer assay conditions were established according to K_d_ and K_p_ values previously obtained. K_d_ was used to ensure low levels of unbound 12-AS (< 5%), and K_p_ to determine SUV:EgAgB molar ratio in order to assure unidirectional transfer to SUVs. EgAgB8/2 or EgAgB8/3 subunits with bound 12-AS were mixed with NBD-PC SUVs. The NBD moiety is an energy transfer acceptor of the anthroyloxy donor group, therefore the fluorescence of the anthroyloxy fatty acid (AOFA) is quenched when the ligand is bound to SUVs which contain NBD-PC. Upon mixing, transfer of AOFA from protein to membrane is directly monitored by the time dependent decrease in anthroyloxy fluorescence. Final transfer assay conditions were 15:1 mol:mol EgAgB:12-AS ratio. SUVs were added ranging from 1:10 mol:mol to 1:40 mol:mol EgAgB:SUVs in TBS buffer at 25°C. Controls to ensure that photobleaching was eliminated were performed prior to each experiment, as previously described [50]. To analyse the influence of membrane surface charge on fatty acid transfer rate, SUVs with 25% negatively charged phospholipids (PS or CL) were employed. Data were analysed employing SigmaPlot software and all curves were well described by a single exponential function. For each experimental condition, at least five replicates were performed. Average values for three separate experiments are reported. Statistical analysis of the data was performed applying one-way analysis of variance (ANOVA) followed by Tukey's Post Hoc Test from GraphPad Prism software.

### Accession numbers

GenBank accession numbers for EgAgB8 subunits: EgAgB8/1: AAD38373, EgAgB8/2: AAC47169, EgAgB8/3: AAK64236, EgAgB8/4: AAQ74958, EgAgB8/5: BAE94835.

## Results and Discussion

### Preparation of lipid-free EgAgB8 subunits

**Fig 2:**
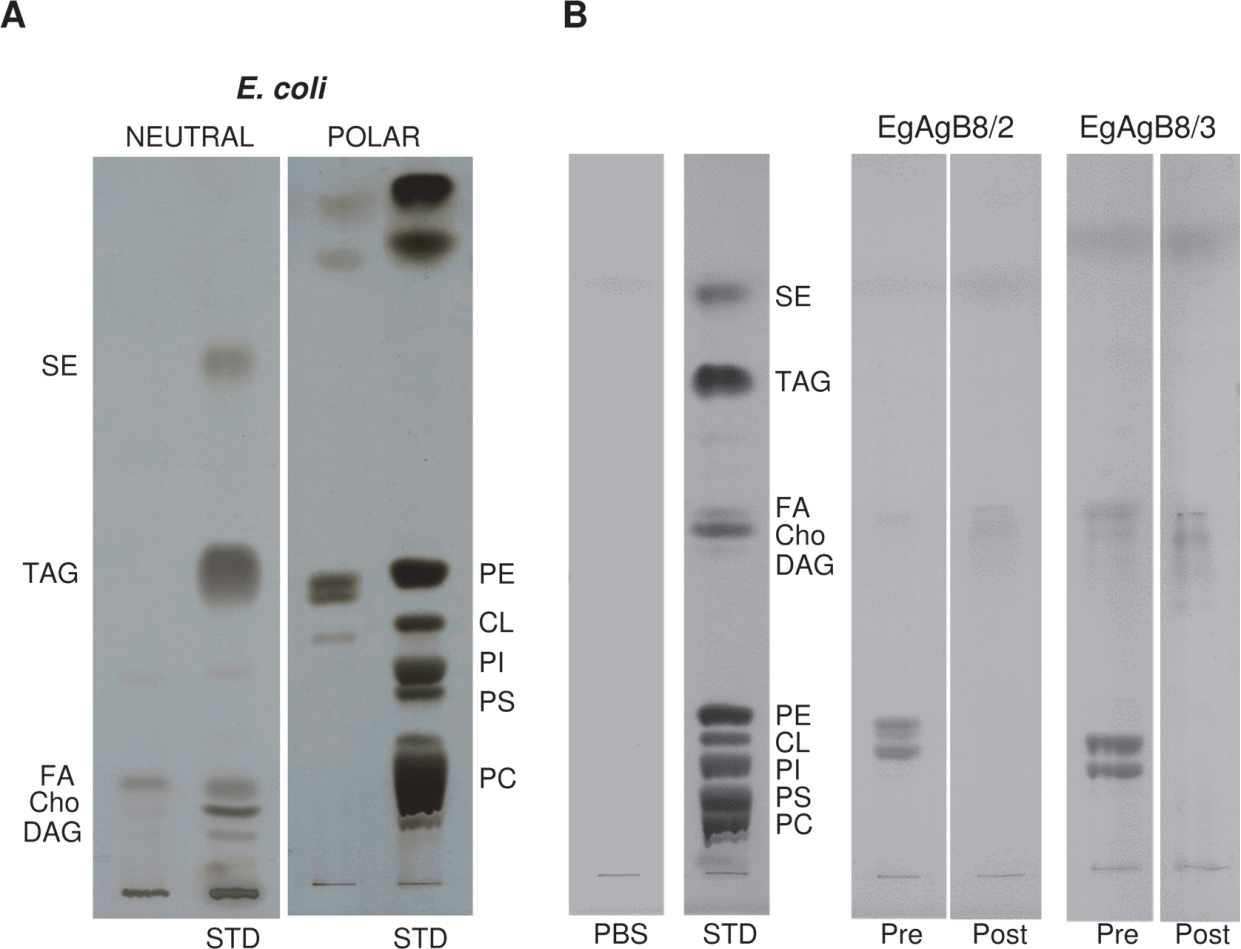
Removal of co-purifying hydrophobic ligands from rEgAgB8 by RP-HPLC. The lipid composition of rEgAgB8 subunits prior to delipidation (Pre), as well as rEgAgB8 subjected to RP-HPLC (Post) were analysed by TLC, in parallel with standards for neutral and polar lipids. Lipid bands were visualised using CuSO_4_/H_3_PO_4_ and identified by comparison with the standards. **(A)** The lipids extracted from *E*. *coli* grown under the same culture conditions is shown for comparison. TLCs from neutral and polar lipids were undertaken separately. **(B)** The lipid moiety of recombinant subunits were analysed by TLC using double development. The lipid fraction of rEgAgB8 pre-HPLC contained mainly polar lipids (PE and CL), which were successfully removed by the RP-HPLC method. PC: phosphatidylcholine; PS: phosphatidylserine; PI: phosphatidylinositol; CL: cardiolipin; PE: phosphatidylethanolamine; Cho: cholesterol; FA: free fatty acids; DAG: diacylglycerols; TAG: triacylglycerols; SE: sterol esters.

We purified lipid-free EgAgB8 subunits in order to analyse their capacity to oligomerize, their lipid binding properties, as well as their ability to transfer fatty acids to membrane vesicles. Among the five distinct EgAgB8 subfamilies, EgAgB8/2 and EgAgB8/3 were chosen because they represent the two distinct subfamilies within EgAgB family as mentioned above (Fig. 1). The recombinant subunits rEgAgB8/2 and rEgAgB8/3 were purified as GST fusion proteins from *E*. *coli* and recovered after thrombin treatment, as previously described [38]. We anticipated that rEgAgB8/2 and rEgAgB8/3 subunits would bind lipids during their synthesis in *E*. *coli*. We therefore analysed by TLC the lipids recovered by Folch extraction from the purified recombinant subunits and compared them with those from *E*.*coli*. Fig. 2A shows that fatty acids and phospholipids, mainly phosphatidylethanolamine (PE) and cardiolipin (CL), are the major lipids present in *E*. *coli* under our extraction conditions. Extractions from both rEgAgB8 subunits yielded mainly PE and CL (Fig. 2B). The absence of phosphatidylcholine, the main phospholipid found in native EgAgB [15], may be due to the lack of this phospholipid in *E*. *coli* (Fig. 2A).

Our next step was to identify a method to efficiently remove bacterial ligands from the proteins. Despite published precedent [35], we found that chromatography with Lipidex 1000 is ineffective at removing lipid from helminth LBPs [39,57,58]. We therefore exploited a method for removal of co-purifying ligands from other recombinant LBPs produced in *E*. *coli* [39], based on RP-HPLC of unfolded protein using a C8-bonded silica and water/acetonitrile/triﬂuoroacetic acid as stationary and mobile phases, respectively. Both rEgAgB8/2 and rEgAgB8/3 subunits were found to bind to C8 column and to elute from this phase at about 70:30 (v/v) acetonitrile/water. After careful refolding employing a large volume of aqueous buffer, the efficacy of this delipidation procedure was assessed by comparing the lipid moiety of treated (post-HPLC) recombinant subunits compared to non delipidated (pre-HPLC) subunits. We found that RP-HPLC successfully removed *E*. *coli* ligands from EgAgB8 subunits (Fig. 2B).

**Fig 3:**
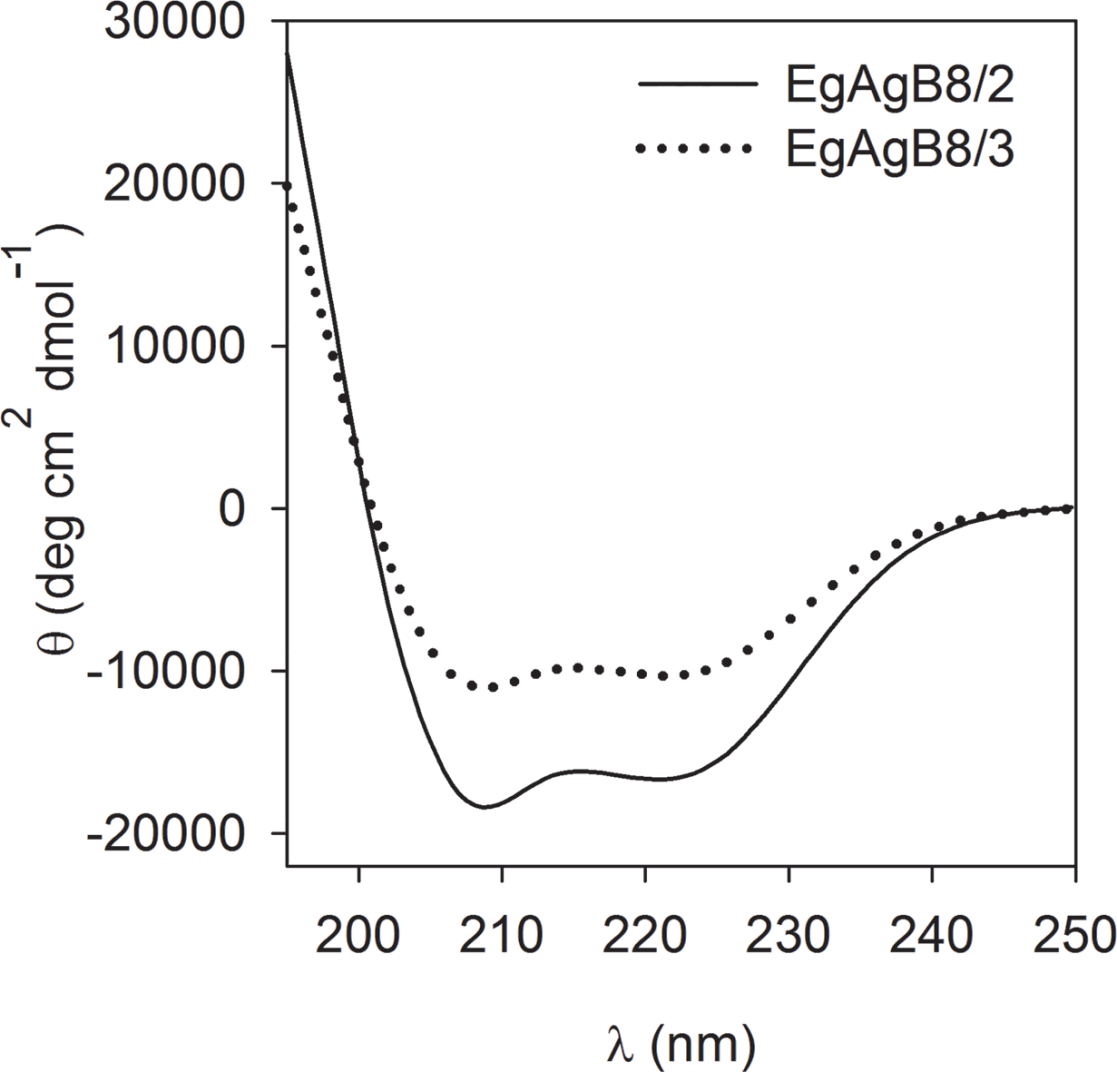
Far UV CD-spectra of lipid-free EgAgB8 subunits. After purification and delipidation, rEgAgB8/2 and rEgAgB8/3 were analysed by CD for secondary structural content. Both subunits showed a predominant alpha-helical structure with double minima at 208 and 222 nm in agreement with their predicted structures [14,33,59]. One representative experiment of two is shown for both EgAgB8 subunits.

In order to check the structural integrity of the proteins after delipidation, we analysed the CD spectra of lipid-free rEgAgB8 subunits after refolding. The spectra of both recombinant subunits presented two minima at 208 and 222 nm, and were consistent with predominantly alpha-helical structures (66% and 30% for rEgAgB8/2 and rEgAgB8/3, respectively) as is shown in Fig. 3. These results are similar to CD data obtained for non-delipidated EgAgB8 subunits (35–40% of alpha-helix [33]) and native EgAgB (between 42 and 65% [14,59]).

Overall, these results showed that the delipidation method based on RP-HPLC provided lipid-free subunits without significant alteration, at least of their secondary structures, permitting us to analyse the interactions of the protein components in isolation.

### Oligomeric states of lipid-free EgAgB8 subunits

**Fig 4:**
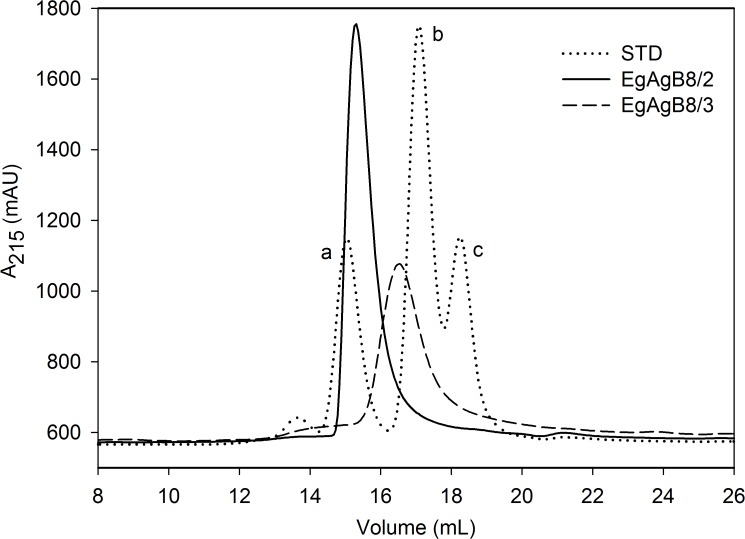
SEC analysis of lipid-free EgAgB subunits. Analysis of EgAgB lipid-free subunits by SEC was undertaken on a Superdex 200 column employing a flow rate of 0.5 mL/min. Elution profiles of lipid-free EgAgB8/2 and EgAgB8/3 were followed at 215 nm. Both subunits showed a well-defined peak, with a molecular weight estimation of 62 kDa for EgAgB8/2 and 38 kDa for EgAgB8/3 according to standard proteins analysed under the same conditions (a: BSA, 66 kDa; b:carbonic anhydrase, 29 kDa; and c: cytochrome c, 12 kDa).

As said, since previous oligomerization studies were performed with non-delipidated proteins, the involvement of lipids in the oligomerization of rEgAgB subunits cannot be ruled out [33,34]. In order to examine whether lipid-free forms are capable of self-associating, we analysed rEgAgB8 subunits by SEC in aqueous solutions. This showed that lipid-free rEgAgB8/2 and rEgAgB8/3 were eluted in defined peaks that were not commensurate with monomeric forms, according to the elution of the standard (Fig. 4). The apparent molecular weight of these subunits indicated the presence of oligomers of 62 and 39 kDa for rEgAgB8/2 and rEgAgB8/3, equivalent to the formation of oligomers of 7–8 subunits and of 4–5 subunits, for lipid-free rEgAgB8/2 and rEgAgB8/3, respectively. Previous reports using non-delipidated recombinant proteins revealed the presence of larger complexes of around 164 kDa for EgAgB8/2 and 113 kDa for EgAgB8/3, as well as secondary peaks of higher molecular mass for both proteins [33]. Nevertheless, our results suggest that oligomerization of EgAgB subunits is not absolutely dependent on the presence of lipids.

**Fig 5:**
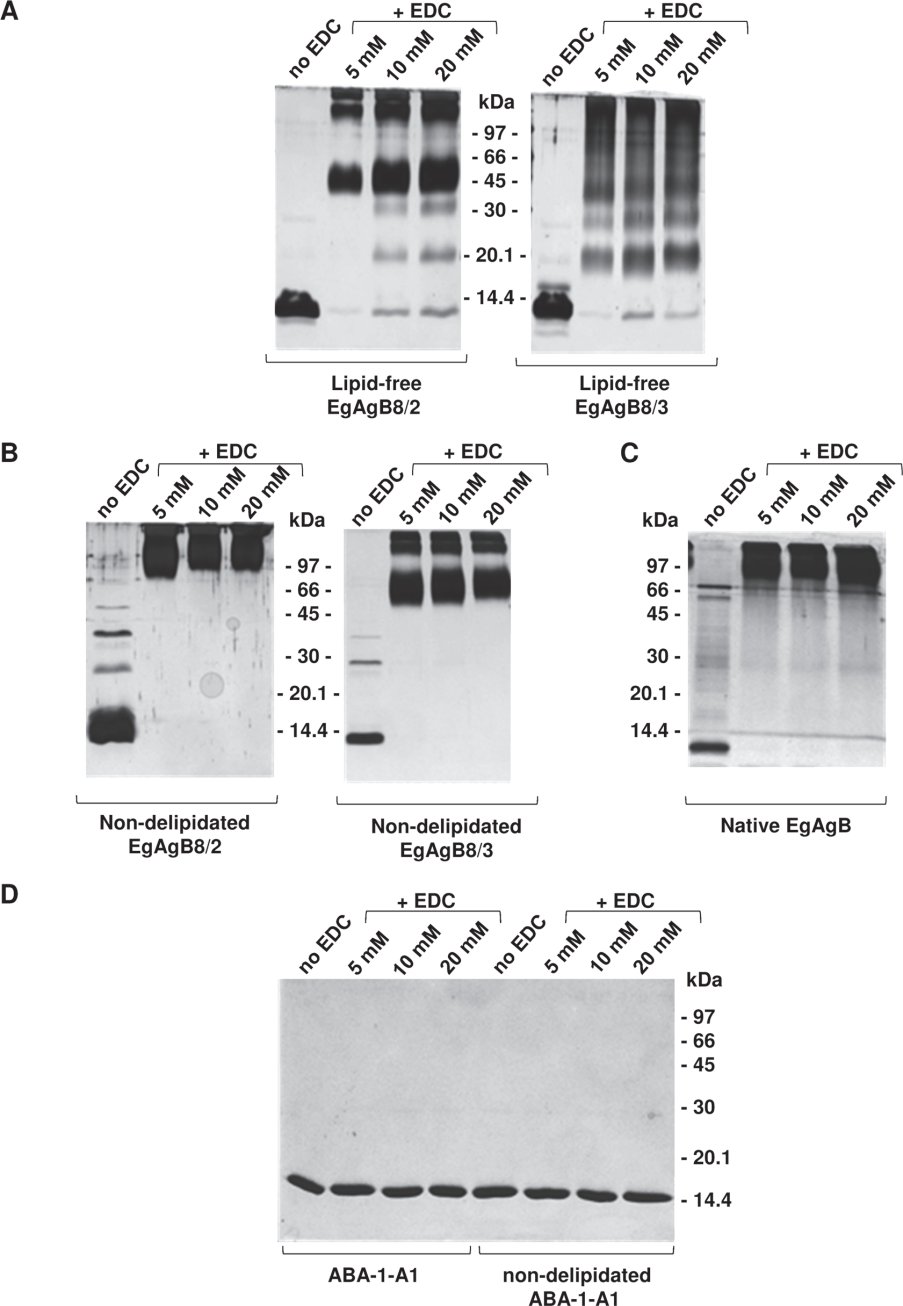
Cross-linking of rEgAgB subunits with EDC. Covalent cross-linking of proteins with EDC was carried out for 30 minutes at 25°C in PBS and separated on 15% SDS-PAGE followed by silver staining. Controls without added EDC were undertaken under the same conditions. **(A)** Cross-linking of lipid-free EgAgB8/2 and EgAgB8/3 subunits. **(B)** Cross-linking of non-delipidated EgAgB8/2 and EgAgB8/3 subunits. **(C)** Cross-linking of native EgAgB. **(D)** Control with lipid-free and non-delipidated ABA-1-A1, a recombinant single unit of the helix-rich LBP of the nematode parasite *Ascaris suum*, which is monomeric at the concentration used. Molecular masses of the standard proteins are indicated in each panel.

In order to further examine the self-assembly of EgAgB8 apolipoproteins, we performed covalent cross-linking experiments analysing the formation of oligomers by SDS-PAGE under reducing conditions (Fig. 5A). We found that in the absence of EDC both subunits were predominantly present as monomers, and EDC addition led to the formation of different cross-linked products. In the range of 5 mM to 20 mM EDC, lipid-free rEgAgB8/2 formed mainly a ∼45 kDa oligomer, whereas lipid-free rEgAgB8/3 formed a heterogeneous array of oligomers (a ladder-like pattern from around 16 to more than 97 kDa). Cross-linked products of higher molecular mass were found for both apolipoproteins. These results suggest that rEgAgB8 subunits have an intrinsic ability to self-assemble, but with differences in the structural organization acquired by rEgAgB8/2 and rEgAgB8/3 in aqueous solution, since the oligomers that they form have different apparent molecular weights according to SEC and EDC cross-linking analysis. For comparison, we also analysed the cross-linked products of non-delipidated rEgAgB8 subunits to assess the role of lipids in EgAgB oligomerization. This showed that non-delipidated proteins form even larger oligomers of apparent MW around 60–90 kDa and higher than 97 kDa (Fig. 5B). These results agree with those previously described by Monteiro and collaborators, obtained using glutaraldehyde as cross-linker [33], and indicate that, whilst lipids are not absolutely required, they may participate in the formation of larger complexes. In parallel, the cross-linking of natural, parasite-derived EgAgB led to the formation of oligomers of high molecular mass, showing a similar pattern to non-delipidated recombinant subunits (Fig. 5C), also in agreement with previous reports [34]. Both, non-delipidated EgAgB subunits and parasite-derived EgAgB, presented larger complexes even in the absence of cross-linker (Fig. 5B and 5C), in contrast to lipid-free EgAgB subunits (Fig. 5A). This suggests that lipids are involved in the oligomerization of EgAgB that is commonly observed in SDS-PAGE analysis under reducing conditions [32]. As a control for cross-linking experiments, we used lipid-free ABA-1-A1 (a helix-rich small LBP from the nematode parasite *Ascaris suum*), a protein which is not expected to oligomerize even at high concentrations [39]. Both lipid-free and non-delipidated ABA-1-A1 were treated under the same conditions and oligomers were not detected (Fig. 5D).

It therefore appears that lipids are not essential for EgAgB subunit self-association, although they may participate in the oligomerization process contributing to the organization of very large EgAgB particles. rEgAgB8/2 and rEgAgB8/3 exhibited different behaviors, although lipid-free rEgAgB8/2 forms an oligomer of greater size than rEgAgB8/3, and rEgAgB8/3 appears to form more heterogeneous oligomers. These results are in concordance with previous findings on non-delipidated rEgAgB8/3 subunits, which formed the more heterogeneous oligomeric states in comparison with rEgAgB8/1 and rEgAgB8/2 [34].

### Binding of fluorescent lipid probes by lipid-free rEgAgB8 subunits

Since the lipid moiety of native EgAgB includes a wide range of lipids, we analysed the capacity of our particular recombinant subunits to bind different fluorescent lipid probes, such as the fatty acid analogues 12-AS and DAUDA, and the cholesterol analogue DHE. These probes have been widely used for characterising the lipid binding properties of LBPs because of their environment-sensitive spectral properties. They have very low fluorescence emission values when free in aqueous solution, but exhibit a significant increase in emission intensity, often accompanied by a blue shift in emission spectrum, when bound to a protein's apolar ligand binding site [60].

**Fig 6:**
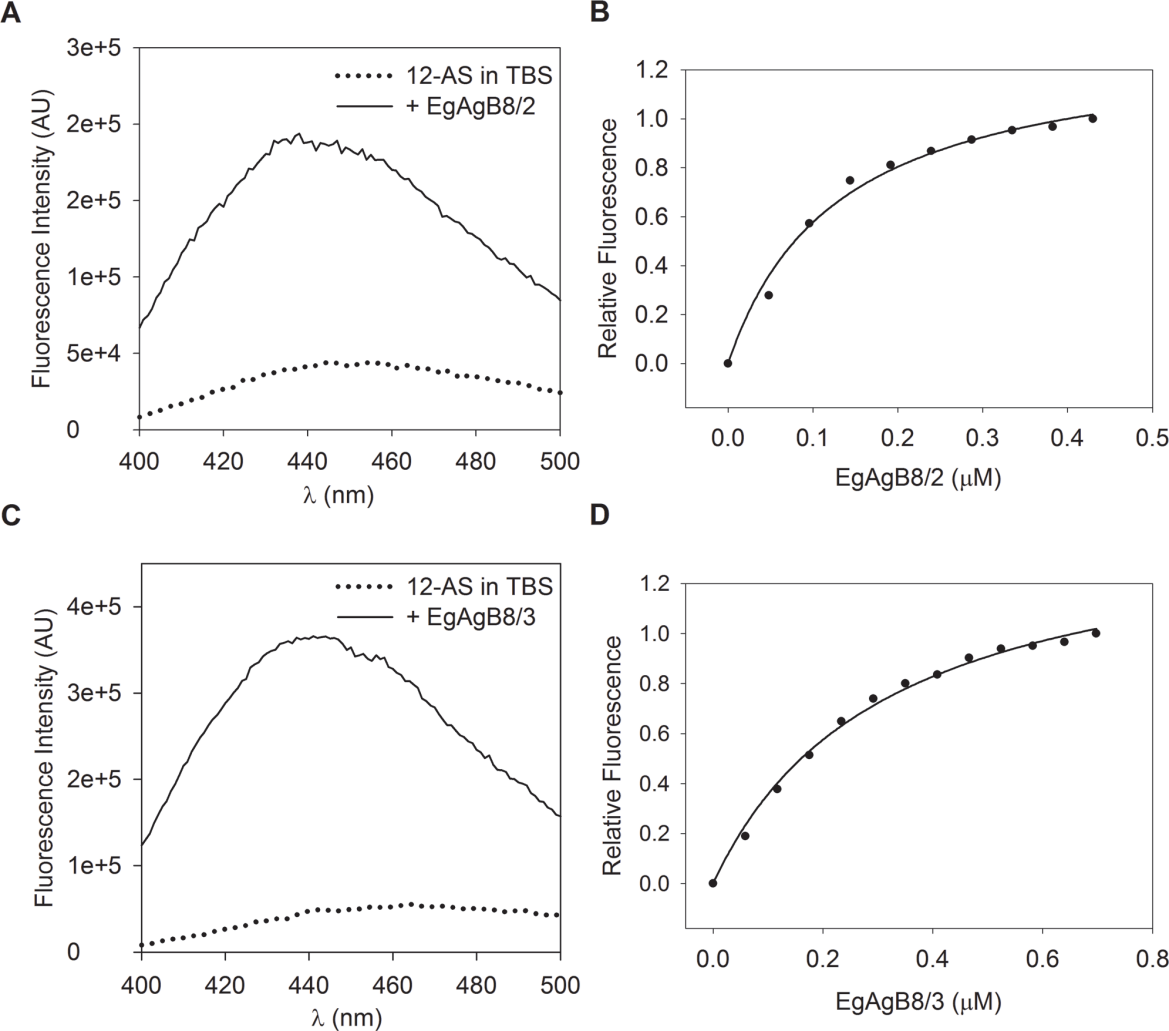
Fluorimetric titration of 12-AS with EgAgB8 subunits. Changes in relative 12-AS fluorescence were monitored from 400 to 500 nm after excitation at 383 nm upon incremental additions of EgAgB8/2 or EgAgB8/3 to a cuvette initially containing 2 mL of 0.5 μM 12-AS in TBS buffer. **(A)** Emission spectra of 12AS in TBS or upon adding EgAgB8/2 (0.5 μM). **(B)** Changes in relative 12-AS fluorescence at 440 nm were used to build the binding isotherm of the complex EgAgB8/2–12AS. **(C)** Emission spectra of 12AS in TBS or upon adding EgAgB8/3 (0.7 μM). **(D)** Changes in relative 12-AS fluorescence at 440 nm were used to build the binding isotherm of the complex EgAgB8/2–12AS. For both proteins, 12-AS spectra showed a blue shift in emission spectrum that accompanies a strong increase in fluorescence emission. The data were consistent with one binding site per monomer unit of protein and K_d_ values of 0.16 ± 0.09 μM for EgAgB8/2 and 0.34 ± 0.02 μM for EgAgB8/3 were obtained using SigmaPlot software. The solid line is the theoretical binding curve for complex formation. One representative experiment of three is shown for both EgAgB8 subunits.

We found that 12-AS showed a significant increase in its fluorescence emission, accompanied by a blue shift from 456 nm to 446 nm when lipid-free rEgAgB8/2 or rEgAgB8/3 subunits were added to the probe solution (Fig. 6A and 6C). For both subunits, titration experiments described curves that reached saturation, in accordance with a ligand binding stoichiometry consistent with 1:1 binding per monomer (Fig. 6B and 6D), with a K_d_ of 0.16 ± 0.09 μM for rEgAgB8/2 (*r = 0*.*9976*) and 0.34 ± 0.02 μM for rEgAgB8/3 (*r = 0*.*9927*). In contrast, negligible enhancement of fluorescent emission was observed for the fatty acid analogue DAUDA upon adding lipid-free rEgAgB8/2 or rEgAgB8/3 (S1 Fig.). Lipidex-treated recombinant subunits EgAgB8/1 and EgAgB8/2 behaved similarly in previous studies since they bound the fatty acid anthroyloxy derivative, 16-AP, but not DAUDA [35]. The K_d_ values determined for the binding of these probes by our delipidated subunits and by Lipidex-treated subunits were similar, but the latter exhibited a lower value for binding sites for monomer (*n* value of approximately 0.3), further emphasising the distinction between methods employed to remove bacterial lipids bound to the recombinant subunits. These findings suggest that the hydrophobic Lipidex resin method is not appropriate to achieve adequate delipidation.

In addition, the fatty acid binding properties of EgAgB subunits could be compared with those of other HLBPs on the basis of the use of similar probes for this analysis, although in these previous studies HLBP delipidation was not reported [36,61,62]. In particular, 16-AP was employed for characterizing the binding capacity of HLBPs, from *Taenia solium* metacestode (TsM150) [36] and *Moniezia expansa* (MeHLBP) [61]. EgAgB subunits seem to be more similar by amino acid sequence to the 10 kDa than to the 7 kDa TsM HLBP subfamily. Moreover members of the former (referred to as recCyDA, recb1 and recm13h) but not of the latter (referred to as RS1) subfamily bind 16-AP [36]. Moreover, recb1 and recm13h were positioned into the same clade with EgAgB, whereas RS1 belonged to a different clade [62]. Furthermore, a recently described HLBP from *T*. *solium*, whose subunits grouped together with MeHLBP rather than with the 7 and 10 kDa TsM HLBP subfamilies, is also capable of binding 16-AP [62]. In the case of the MeHLBP monomers, the binding is consistent with a stoichiometry value of 1:1, and a K_d_ value of 2 μM, suggesting that lipid-free rEgAgB8 subunits exhibit a higher affinity for AOFA probes [61]. Taken together, these results indicate that various HLBPs are potentially capable of binding fatty acids, and that differences in their ligand binding properties are not easy to predict from their presumed evolutionary relationships.

On the other hand, the fluorescent cholesterol analogue, DHE, was found not to be bound by neither rEgAgB8/2 nor rEgAgB8/3 (S2 Fig.), suggesting that lipid-free EgAgB8/2 and EgAgB8/3 subunits do not directly interact with cholesterol. The presence of cholesterol in EgAgB purified from hydatid cyst fluid [15], suggests that it may be incorporated into the particle once EgAgB subunits have already bound their lipid ligands. On the other hand, the interaction of cholesterol with other EgAgB subunits cannot be ruled out.

In order to employ natural ligands instead of fluorescent ones we evaluated the effect of cholesterol and oleic acid on the intrinsic rEgAgB8/2 fluorescence using Trp16 as a reporter. This methodology has been successfully used for other HLBP members [61,63] and could not be applied to rEgAgB8/3 characterisation since this subunit does not contain Trp residues. Changes in Trp16 fluorescence were not observed when cholesterol or oleic acid were added to lipid-free rEgAgB8/2, indicating that the environment of Trp16 was not sensitive to the binding of these lipids (S3 Fig.). These results agree with previous observations obtained using Lipidex-treated rEgAgB8/2 and natural fatty acids as ligands [35], and suggest that Trp16 is not close to the putative ligand binding site for fatty acids present in this subunit.

Overall, our results, together with previous data, suggest that lipid-free rEgAgB8/2 and rEgAgB8/3 apolipoproteins have a selective capacity to bind lipids, showing affinity at least for 16- and 18-C fatty acids, but not for cholesterol, indicating that these components of the natural EgAgB lipoprotein particles would not interact directly with cholesterol.

### Transfer of fluorescent fatty acids from EgAgB8 subunits to artificial phospholipid membranes

**Fig 7:**
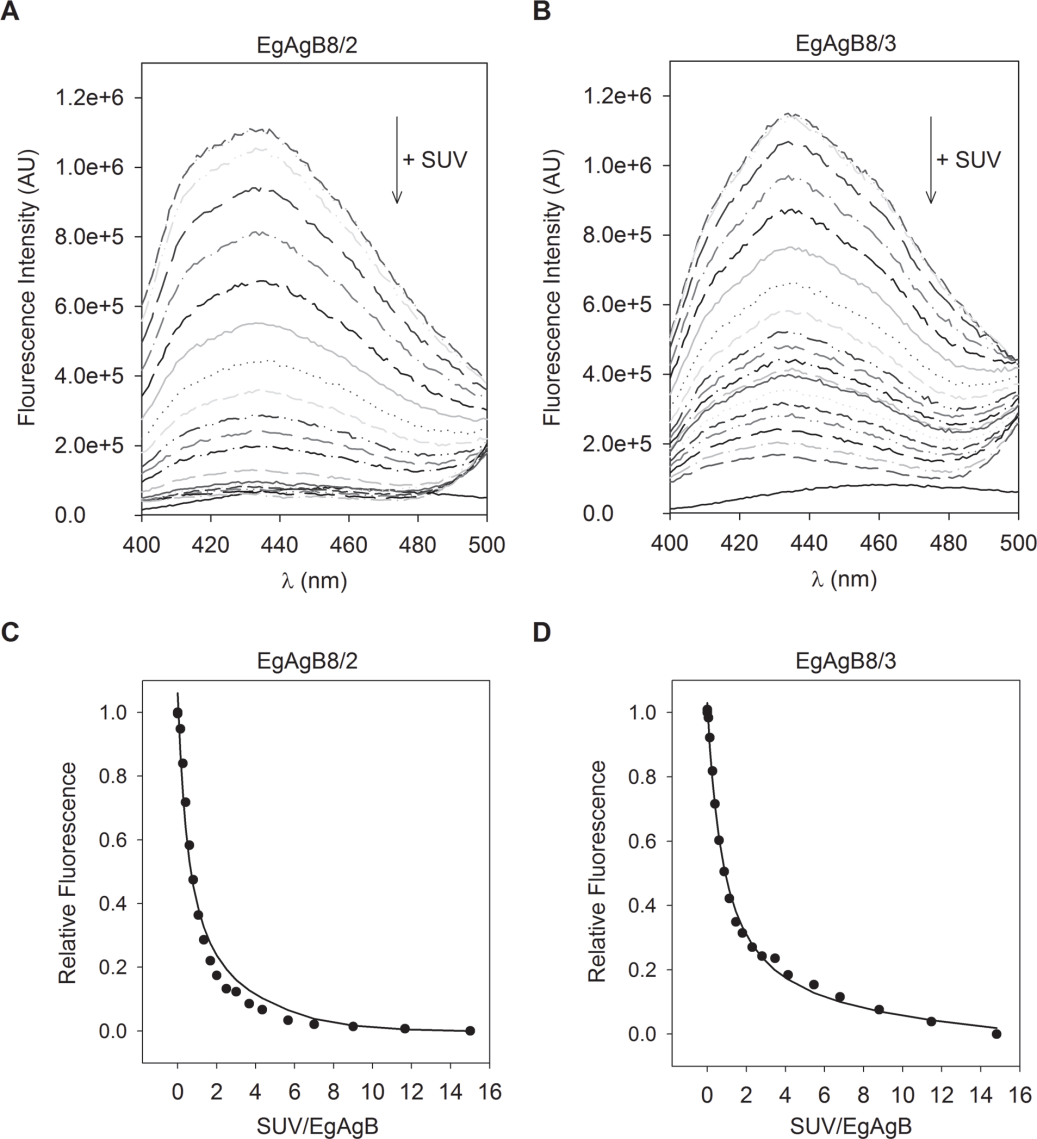
Equilibrium partition of 12-AS between EgAgB8 subunits and SUVs. The K_p_ for 12-AS partitioning was determined by measuring 12-AS fluorescence after addition of SUVs into a solution containing 7.5 μM EgAgB8/2 or EgAgB8/3 and 0.5 μM 12-AS in buffer TBS at 25°C (15:1 mol:mol). **(A)**, **(B)** Emission spectra changes of 12AS bound to EgAgB8/2 or EgAgB8/3 respectively, upon incremental addition of EPC-SUVs containing NBD-PC. **(C)**, **(D)** Changes in relative 12-AS fluorescence were used to obtained K_p_ values, fitting the Equation 2 described in Materials and Methods to the data, employing *Solver Add-In* from Excel (solid line). K_p_ values of 0.62 ± 0.09 and 0.88 ± 0.15 were obtained for rEgAgB8/2 and rEgAgB8/3, respectively. One representative experiment of two is shown for both EgAgB8 subunits.

**Fig 8:**
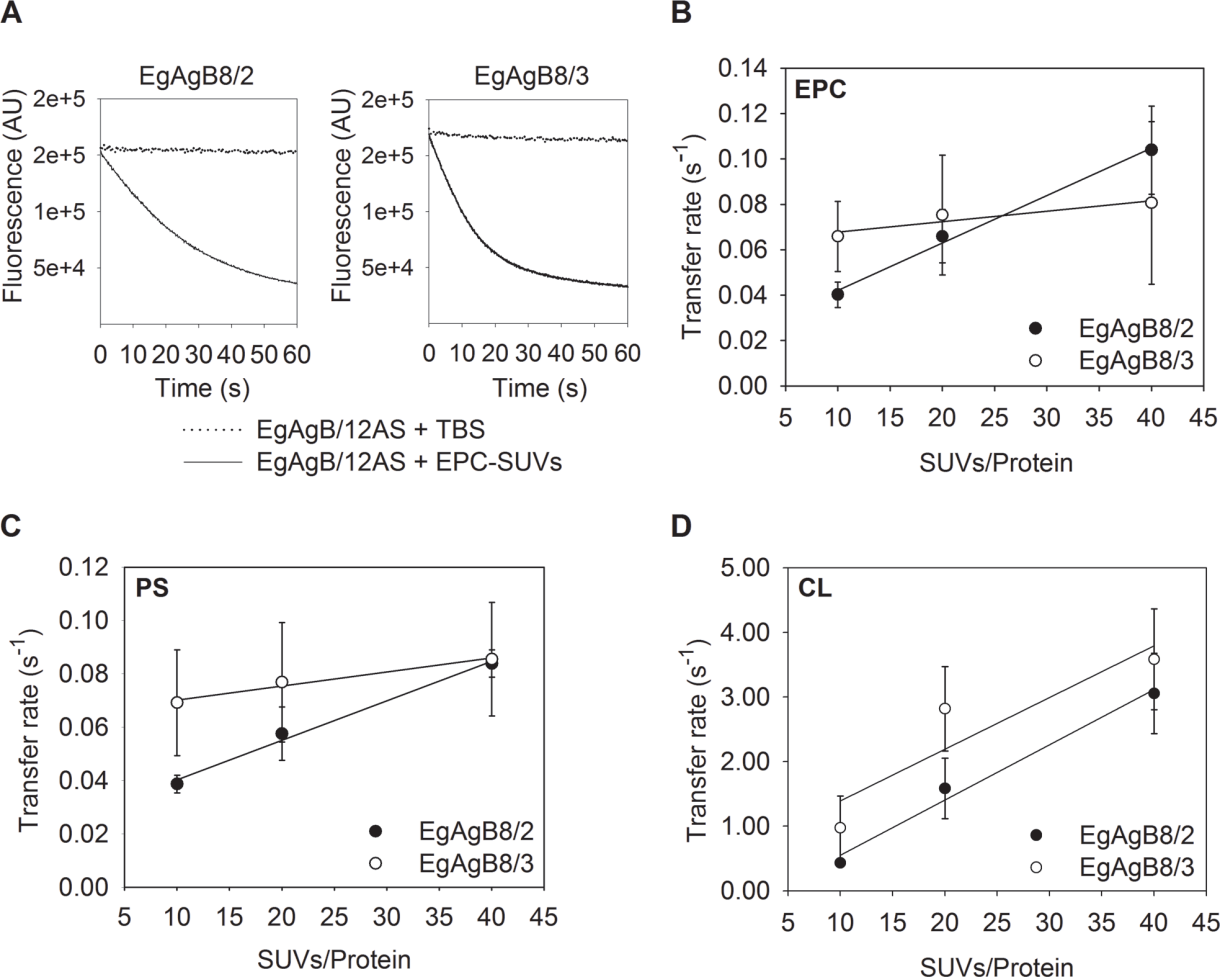
Effect of acceptor membrane concentration on 12-AS transfer from EgAgB8/2 and EgAgB8/3 to different SUVs. Transfer of 12-AS from EgAgB8/2 or EgAgB8/3 to SUVs was monitored by adding SUVs in a molar ratio of 10:1, 20:1 and 40:1 (SUVs/Protein) to the complex EgAgB8/2:12AS or EgAgB8/3:12AS (15:1 mol:mol). **(A)** Representative kinetic trace obtained when combining EgAgB8–12AS with NBD-PC-containing vesicles (molar ratio SUV/Protein of 10:1). Photobleaching control adding TBS instead of NBD-PC/SUVs is shown. **(B)** SUVs containing 100% EPC; **(C)** 75% EPC, 25% PS or **(D)** 75% EPC, 25% CL were used. For each experimental condition at least five replicates of the kinetic curves were done. All curves were well-described by a single exponential function to obtain each transfer rate value employing SigmaPlot software. Transfer rates (mean ± SD) of three independent experiments are reported. Statistical analysis of the data was carried out employing ANOVA followed by Tukey's Post Hoc Test from GraphPad Prism software.

How lipids carried by EgAgB complexes may be distributed within the parasite is unknown, so, we attempted to characterise the capacity of these subunits to transfer lipids to membranes. Since fatty acids, but not cholesterol, were bound by delipidated rEgAgB8 subunits, assays were designed to examine the transfer of 12-AS to phospholipid artificial membranes (SUVs). To establish the conditions for the transfer measurements we used the K_d_ values obtained for rEgAgB8/2 and rEgAgB8/3 to ensure that less than 5% of 12-AS remained free in solution. We also determined the K_p_ of 12-AS between rEgAgB8 subunits and vesicles to assure unidirectional transfer of 12-AS from rEgAgB8 to the vesicles. In order to do this, SUVs containing a FRET acceptor of the anthroyloxy group donor (NBD-PC) were added to a solution of 12-AS:EgAgB8/2 or 12-AS:EgAgB8/3 complex. The 12-AS fluorescence decay upon incremental increase in SUV concentration for both 12-AS:EgAgB8 complexes is shown in Fig. 7, indicating the transfer of 12-AS from EgAgB8 subunits to NBD-PC-containing vesicles. Using Eq. 2 (see Materials and Methods) a K_P_ value of 0.62 ± 0.09 was obtained for rEgAgB8/2 and of 0.88 ± 0.15 for rEgAgB8/3. Both indicate that there is a slightly higher preference of 12-AS for the phospholipid membranes. Once these conditions were established, we analysed the transfer rates of 12-AS bound to rEgAgB8/2 or rEgAgB8/3 to the vesicles. Firstly we examined the transfer rates as a function of SUV concentration to determine whether the limiting step for ligand transfer is the effective protein-membrane interaction or the dissociation of the protein-ligand complex, as has been previously established for other LBPs [49–56]. A representative time trace of 12-AS fluorescence change upon SUV addition to EgAgB8/2:12-AS or EgAgB8/3:12-AS complexes is shown in Fig. 8A. We found that the 12-AS transfer rate from rEgAgB8/2 to EPC-SUVs increased significantly from 0.039 ± 0.003 s^−1^ to 0.084 ± 0.005 s^−1^ (*p* < 0.05) when SUV:protein ratio raised from 10:1 to 40:1. In the case of rEgAgB8/3, a trend towards an increase in 12-AS transfer rate (from 0.07 ± 0.02 s^−1^ to 0.08 ± 0.02 s^−1^) was observed, but this trend did not reach statistical significance (Fig. 8B). These results using zwitterionic SUVs suggest that the mechanism of 12-AS ligand transfer differed between EgAgB subunits; for rEgAgB8/2 the limiting step for transfer is the direct contact with the vesicle (collisional mechanism), whereas for rEgAgB8/3 the dissociation of 12-AS from the complex seems to be the limiting step (diffusional mechanism).

We then proceeded to examine whether electrostatic interactions between EgAgB subunits and SUVs could affect the ligand transfer mechanism. For this purpose we carried out similar transfer rate assays using negatively charged vesicles, which were obtained by incorporating PS or CL into the acceptor vesicles (one net negative charge per molecule of PS, two net negative charges per molecule of CL). Using PS-SUVs (Fig. 8C), 12-AS transfer rates of both rEgAgB8 subunits were similar to those observed for zwitterionic SUVs. Once again, the 12-AS transfer rate showed a statistically significant increase for rEgAgB8/2, but not for rEgAgB8/3 when the SUV:protein ratio exhibited a 4-fold increase. This suggests that the transfer of 12-AS to phospholipid membranes by EgAgB8 apolipoproteins was not significantly modified by the increase in the negative charge of the vesicles caused by PS incorporation. In contrast, when CL-SUVs were used, 12-AS transfer rates of both EgAgB8 proteins were remarkably increased. Moreover, for both EgAgB8 subunits, the transfer rates reached higher values at higher SUV:protein ratios (Fig. 8D), ranging from 0.43 ± 0.03 s^−1^ to 3.1 ± 0.6 s^−1^ for rEgAgB8/2 and from 0.9 ± 0.5 s^−1^ to 3.6 ± 0.6 s^−1^ for rEgAgB8/3. These results indicate that the greater increase in the negative charge of SUVs caused by CL incorporation enhanced the ability of rEgAgB8/2 and rEgAgB8/3 to transfer their ligands to vesicles, via a collision-mediated mechanism. Overall, these results suggest that electrostatic interactions between EgAgB8 subunits and phospholipid membranes are of foremost importance for determining the rate at which these proteins can transfer their ligands. Nevertheless, an increase in the affinity of EgAgB8 subunits to CL-SUVs could not be ruled out. Furthermore, the greater ability of rEgAgB8/2 to transfer 12-AS to CL-SUV vesicles than rEgAgB8/3 (*p* < 0.05, S4 Fig.) may be related to the negative and positive charge distribution in these proteins [16].

This report provides new approaches towards an understanding of cestodes HLBPs and how they may interact with phospholipid membranes to transfer their ligands. Since there are no reports for other HLBP members, EgAgB lipid transfer properties can only be compared to those of other LBP families [49–56]. As mentioned above, although the mechanisms employed by EgAgB8/2 and EgAgB8/3 to transfer 12-AS to EPC vesicles appear to be different, in both cases transfer rates are of the same order of magnitude. Previous studies have shown that LBPs that exchange ligands by collisional or diffusional processes also differ in the range of rates employed to transfer their ligands to acceptor membranes. For example, mammalian intestinal fatty acid binding protein (IFABP) and *Schistosoma japonicum* FABP (Sj-FABPc), both employing collisional mechanisms, transfer their ligands at a higher rate compared to diffusional proteins such as liver FABP and ABA-1-A1 protein from *A*. *suum* [49,51]). Furthermore, for proteins that transfer ligands through a collision-mediated mechanism, transfer rates increase proportionally to vesicle concentration (e.g. intestinal FABP, Sj-FABPc, EgFABP1 from *E*. *granulosus* or YLSCP2 from the yeast *Yarrowia lipolytica* [49,51,55,56]), and this is the case for EgAgB8/2. On the other hand, diffusional proteins have not shown variations in transfer rates as vesicle concentration or vesicle composition changes (e.g., liver-FABP, ABA-1-A1 or Ov-FAR-1 protein from *Onchocerca volvulus* [49,51]). Remarkably, the EgAgB8/3 transfer rate of 12-AS does not change with increasing concentrations of EPC or PS-SUVs, but a significant increase was observed with CL-SUVs, suggesting EgAgB8/3 employs a mechanism that is different to those described so far. Overall, a comparison of our results with those reported for other LBPs suggests that while EgAgB8/2 behaves as a typical collisional protein, EgAgB8/3 does not, even compared to other α-helical proteins such as ABA-1-A1, Ov-FAR-1 or YLSCP2. Whether this is an exclusive feature of EgAgB8/3 subunit or is a conserved behaviour of certain subunits of other HLBPs, needs further investigation.

Finally, our results showed that EgAgB8/2 and EgAgB8/3 subunits are able to deliver their cargo to phospholipid membranes, supporting the hypothesis that EgAgB is involved in lipid transport between parasite and host tissues [16]. Nevertheless, the capacity of EgAgB particles to transfer fatty acids to the parasite or to the host´s cells remains to be formally demonstrated.

### Concluding remarks

The small proteins that collectively form the large Antigen B complexes present in the hydatid cysts of *Echinococcus* spp. and the similar entities in the cysticerci of other highly pathogenic cestodes, comprise the most abundant proteins present in the fluids of these parasites. Their native structures and role in lipid dynamics of the parasites remain to be elucidated, a difficulty being that our understanding of how the various components of the large Antigen B complexes form, what ligands they bind, and how, is at best rudimentary. In particular, the separate or synergistic roles of the small protein isoforms and lipid components remain mysterious. The advance that we report here is to provide for the first time a method for the complete removal of lipids from single recombinant isoforms of the protein components without detectably compromising their structures or biochemical activities, and the demonstration that self-assembly of complexes does not absolutely depend on lipids. It still appears, however, that lipids may enhance the process for the formation of the large complexes found in the natural product. In the natural particles, the complexes comprise heterogeneous mixtures of several different isoforms of the EgAgB proteins, with different types of lipids [15]. We have therefore provided the basis for the analysis of self-assembly that should eventually permit elucidation of potentially cooperative interactions of EgAgB isoforms and lipids. In early studies it has been suggested that EgAgB subunits are elongated and amphipathic molecules which could form multimeric structures thermodynamically more stable than individual monomers [33]. Recently, the prediction of the tertiary structure of EgAgB subunits suggests that the position of hydrophilic and hydrophobic amino acids defines pocket-like regions where hydrophobic ligands could interact with the proteins, and a partial charge distribution showing a plausible electrostatic profile for molecular oligomerization [16], in concordance with the experimental data we obtained in this work. Based on the information obtained from molecular modelling studies we can now begin to identify the motifs in EgAgB apolipoproteins that are involved in oligomerization or ligand binding.

The developments we report also allowed us to address the question of how EgAgB complexes may interact with cell membranes of the parasite in order to acquire, transfer and deliver lipids within the parasites. We found that they can deliver their cargo to phospholipid membranes through direct physical interaction with them, and that the charge composition of the membranes is crucial to this process. The way is now open to examine such processes using parasite cell lines or miniature hydatid cysts which are now available [64–67].

It has been more than 40 years since EgAgB was described as an abundant lipoprotein in the hydatid cyst fluid [13], and it has been successfully employed as an *Echinococcus* genus-specific target antigen for human serodiagnosis [19–22]. We know that lipids, such as cholesterol and fatty acids, are essential for *E*. *granulosus* s. l. [18] and that EgAgB is able to bind them *in vivo* [15]. Thus, EgAgB’s unusual construction and its participation in lipid uptake and delivery, together with the fact that it has no homologue in other animal phyla, places it in an excellent position to be seriously considered for therapeutic intervention. Our findings on EgAgB’s unusual lipid-binding, self-assembly, and membrane interaction properties therefore potentially present new avenues for drug developments that could disable a range of physiological processes essential to the parasite’s establishment and survival such as membrane construction and lipid-based signalling systems.

## Supporting Information

S1 Fig Fluorimetric titration of DAUDA with EgAgB8 subunits. Changes in relative DAUDA fluorescence were monitored from 365 to 665 nm after excitation at 345 nm upon incremental additions of EgAgB8/2 or EgAgB8/3 to a cuvette initially containing 2 mL of 0.5 μM DAUDA in TBS buffer. (A) Emission spectra of DAUDA in TBS (dotted line) or upon adding the highest EgAgB8/2 concentration employed (2 μM, solid line). (B) Emission spectra of DAUDA in TBS (dotted line) or upon adding the highest EgAgB8/3 concentration employed (2 μM, solid line). One representative experiment of two is shown for both EgAgB8 subunits.
(TIF)

S2 Fig Fluorimetric titration of DHE with EgAgB8 subunits. Changes in relative DHE fluorescence were monitored from 350 to 515 nm after excitation at 325 nm upon incremental additions of EgAgB8/2 or EgAgB8/3 to a cuvette initially containing 2 mL of 0.5 μM DHE in TBS buffer. (A) Emission spectra of DHE in TBS (dotted line) or upon adding the highest EgAgB8/2 concentration employed (2.6 μM, solid line). (B) Emission spectra of DHE in TBS (dotted line) or upon adding the highest EgAgB8/3 concentration employed (2.6 μM, solid line). One representative experiment of two is shown for both EgAgB8 subunits.
(TIF)

S3 Fig Ligand binding analysis employing Trp16 fluorescence of EgAgB/2. Changes in relative Trp16 fluorescence of EgAgB8/2 were monitored from 310 to 400 nm after excitation at 295 nm. (A) Emission spectra changes in Trp16 fluorescence upon incremental additions of oleic acid to a cuvette initially containing 2 mL of 5 μM EgAgB8/2 in TBS buffer (0.5, 1.0, 1.5 y 6.5 μM). (B) Emission spectra changes in Trp16 fluorescence upon incremental additions of cholesterol to a cuvette initially containing 2 mL of 2 μM EgAgB8/2 in TBS buffer (0.7, 1.4, 2.2, 3.6, 5.1 y 19.4 μM). One representative experiment of two is shown.
(TIF)

S4 Fig Effect of acceptor membrane surface charge on 12-AS transfer from EgAgB8/2 and EgAgB8/3 to SUVs. To compare the transfer rates of 12-AS from EgAgB8/2 or EgAgB8/3 to SUVs with different negative surface charges, we used SUVs containing 100% EPC; 75% EPC, 25% PS (single negative charge) or 75% EPC, 25% CL (double negative charge) as detailed in the main text. Molar ratios of 20:1 (SUVs/Protein) and 15:1 mol:mol of the complex EgAgB8/2:12AS or EgAgB8/3:12AS were used for this comparison. For each experimental condition at least five replicates of the kinetic curves were done. All curves were well-described by a single exponential function to obtain each transfer rate value using SigmaPlot software. Transfer rates (mean ± SD) of three independent experiments for EgAgB8/2 (dark grey bars) and EgAgB8/3 (light grey bars) are reported. Statistical analysis of the data was carried out employing ANOVA followed by Tukey's Post Hoc Test from GraphPad Prism software.
(TIF)
