# Supplementary figures and images for: Lipid-Free Antigen B Subunits from Echinococcus granulosus: Oligomerization, Ligand Binding, and Membrane Interaction Properties
Source: PLoS Negl Trop Dis. 2015 Mar 13;9(3):e0003552. doi: 10.1371/journal.pntd.0003552 (PMC4358968; doi:10.1371/journal.pntd.0003552)

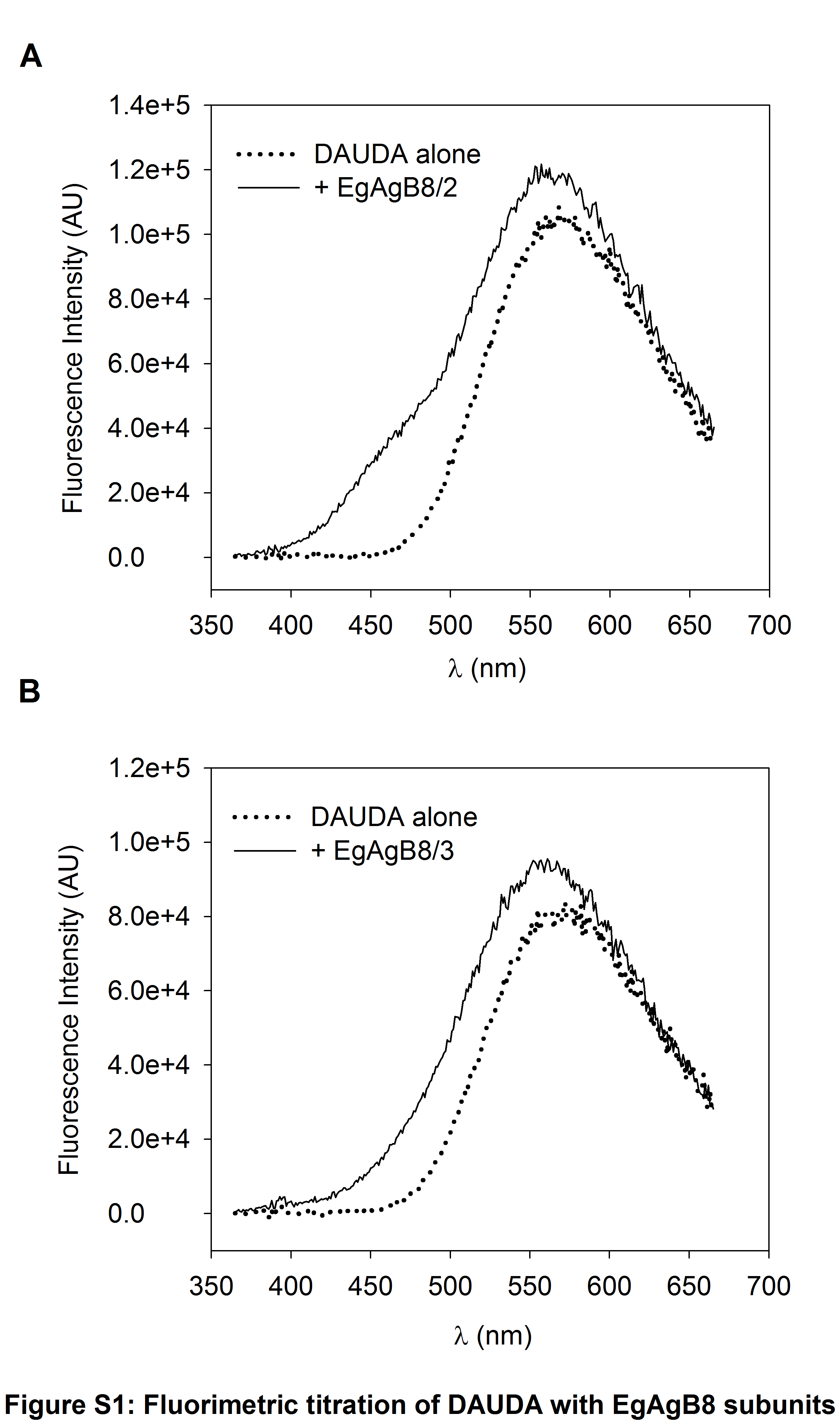

Supplement: S1 Fig — Changes in relative DAUDA fluorescence were monitored from 365 to 665 nm after excitation at 345 nm upon incremental additions of EgAgB8/2 or EgAgB8/3 to a cuvette initially containing 2 mL of 0.5 μM DAUDA in TBS buffer. (A) Emission spectra of DAUDA in TBS (dotted line) or upon adding the highest EgAgB8/2 concentration employed (2 μM, solid line). (B) Emission spectra of DAUDA in TBS (dotted line) or upon adding the highest EgAgB8/3 concentration employed (2 μM, solid line). One representative experiment of two is shown for both EgAgB8 subunits. (TIF) [file pntd.0003552.s001.TIF]

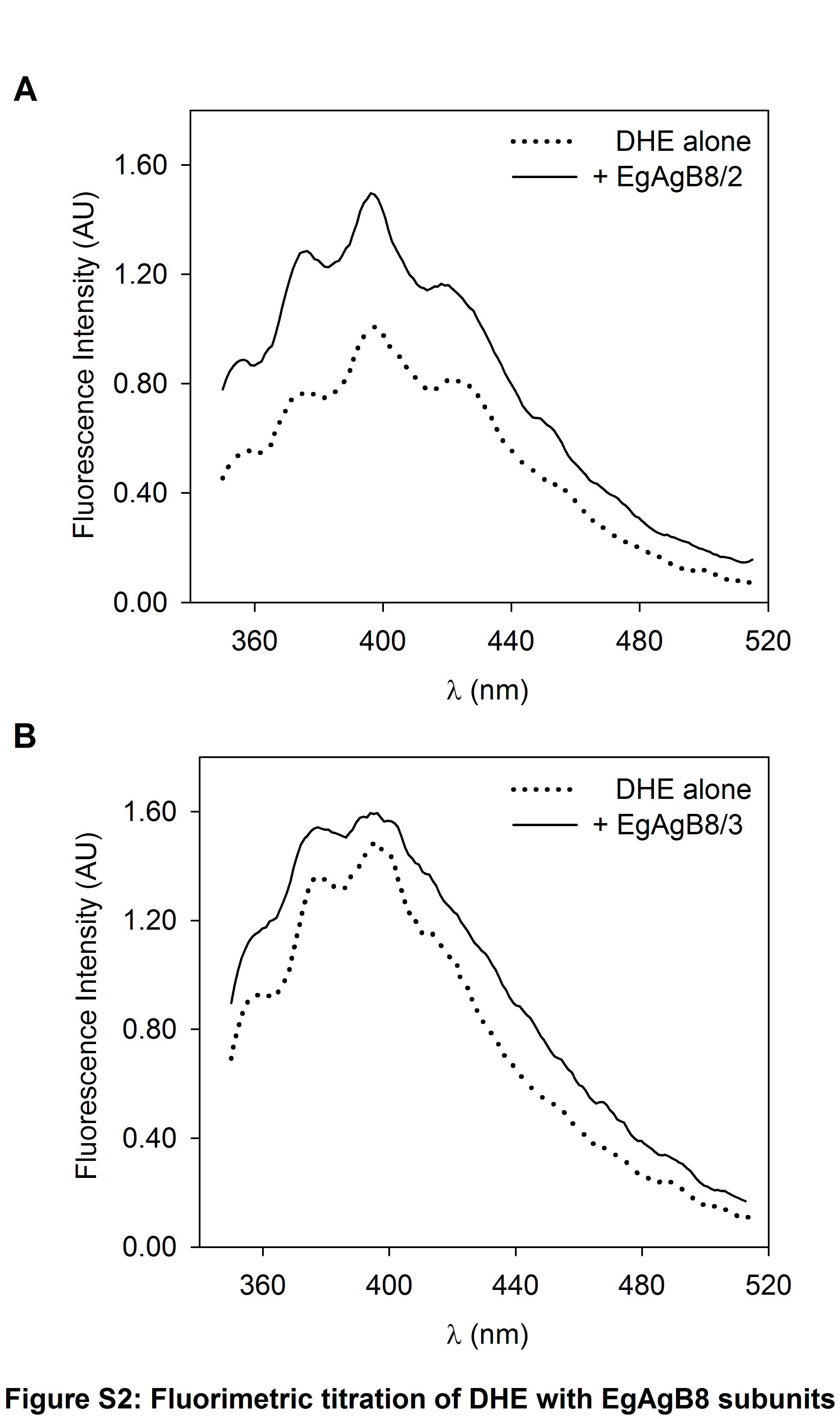

Supplement: S2 Fig — Changes in relative DHE fluorescence were monitored from 350 to 515 nm after excitation at 325 nm upon incremental additions of EgAgB8/2 or EgAgB8/3 to a cuvette initially containing 2 mL of 0.5 μM DHE in TBS buffer. (A) Emission spectra of DHE in TBS (dotted line) or upon adding the highest EgAgB8/2 concentration employed (2.6 μM, solid line). (B) Emission spectra of DHE in TBS (dotted line) or upon adding the highest EgAgB8/3 concentration employed (2.6 μM, solid line). One representative experiment of two is shown for both EgAgB8 subunits. (TIF) [file pntd.0003552.s002.TIF]

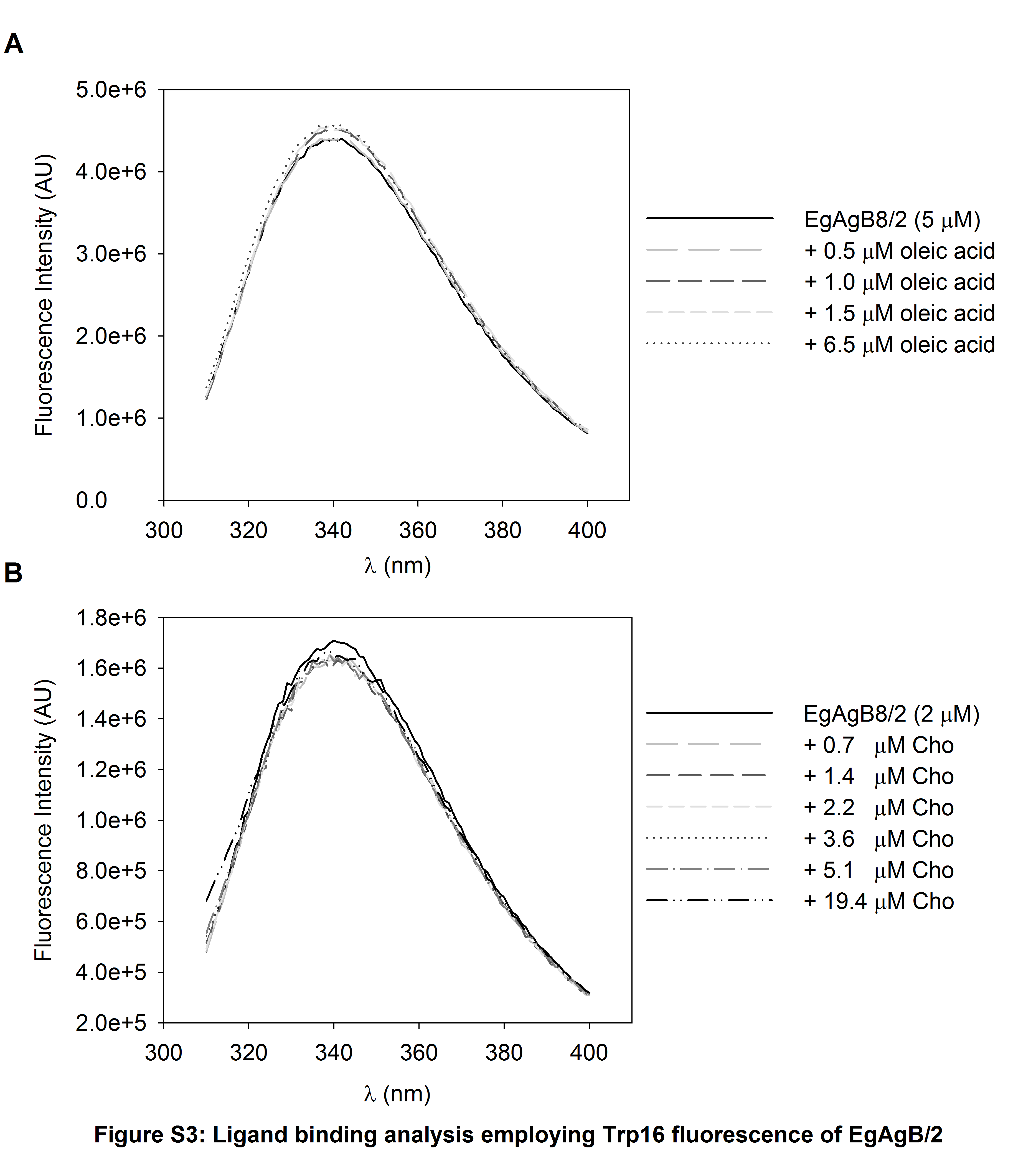

Supplement: S3 Fig — Changes in relative Trp16 fluorescence of EgAgB8/2 were monitored from 310 to 400 nm after excitation at 295 nm. (A) Emission spectra changes in Trp16 fluorescence upon incremental additions of oleic acid to a cuvette initially containing 2 mL of 5 μM EgAgB8/2 in TBS buffer (0.5, 1.0, 1.5 y 6.5 μM). (B) Emission spectra changes in Trp16 fluorescence upon incremental additions of cholesterol to a cuvette initially containing 2 mL of 2 μM EgAgB8/2 in TBS buffer (0.7, 1.4, 2.2, 3.6, 5.1 y 19.4 μM). One representative experiment of two is shown. (TIF) [file pntd.0003552.s003.TIF]

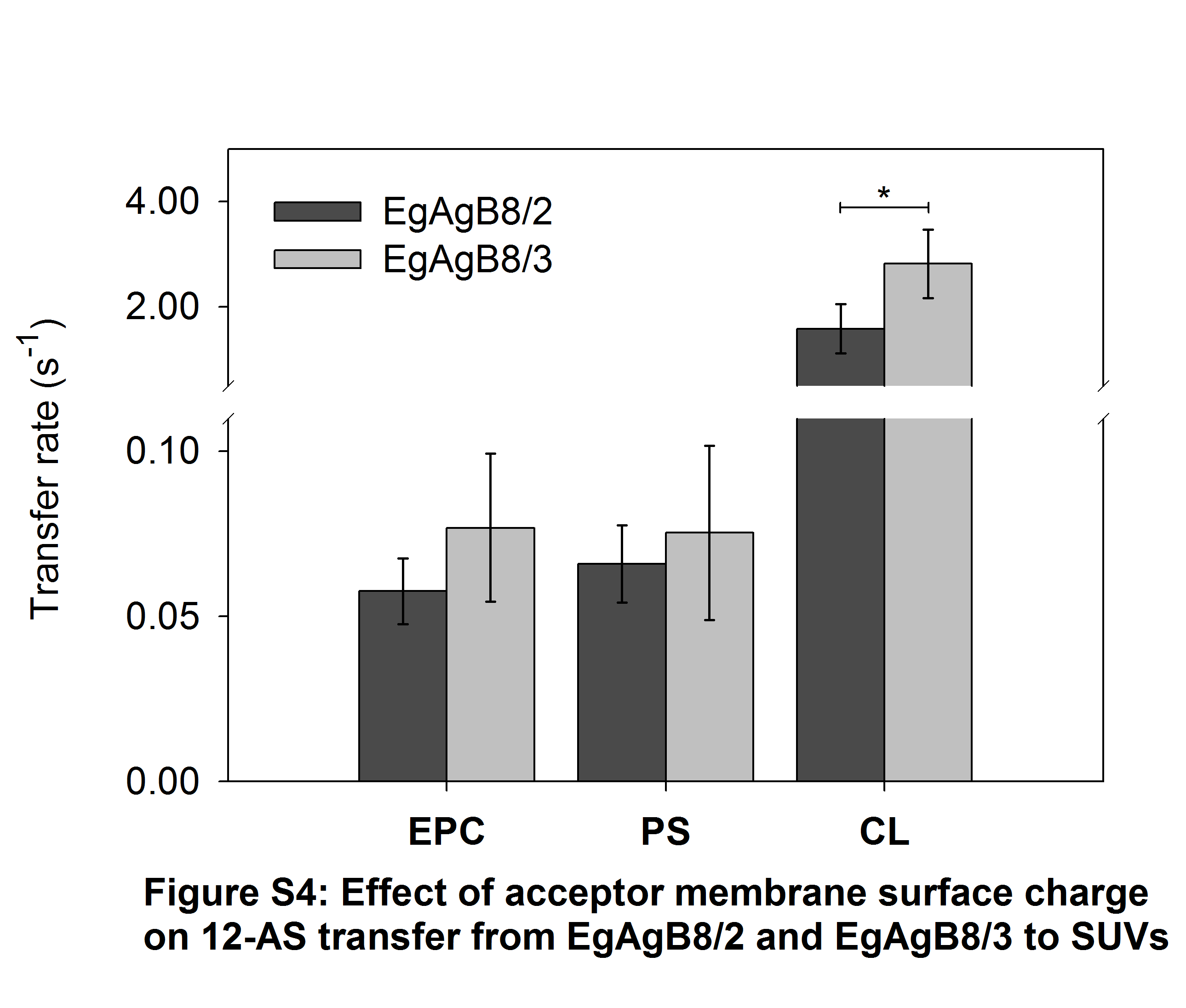

Supplement: S4 Fig — To compare the transfer rates of 12-AS from EgAgB8/2 or EgAgB8/3 to SUVs with different negative surface charges, we used SUVs containing 100% EPC; 75% EPC, 25% PS (single negative charge) or 75% EPC, 25% CL (double negative charge) as detailed in the main text. Molar ratios of 20:1 (SUVs/Protein) and 15:1 mol:mol of the complex EgAgB8/2:12AS or EgAgB8/3:12AS were used for this comparison. For each experimental condition at least five replicates of the kinetic curves were done. All curves were well-described by a single exponential function to obtain each transfer rate value using SigmaPlot software. Transfer rates (mean ± SD) of three independent experiments for EgAgB8/2 (dark grey bars) and EgAgB8/3 (light grey bars) are reported. Statistical analysis of the data was carried out employing ANOVA followed by Tukey's Post Hoc Test from GraphPad Prism software. (TIF) [file pntd.0003552.s004.TIF]
